# CT-Angiography–Based Evaluation of the Aortic Annulus for Prosthesis Sizing in Transcatheter Aortic Valve Implantation (TAVI)–Predictive Value and Optimal Thresholds for Major Anatomic Parameters

**DOI:** 10.1371/journal.pone.0103481

**Published:** 2014-08-01

**Authors:** Florian Schwarz, Philipp Lange, Dominik Zinsser, Martin Greif, Peter Boekstegers, Christoph Schmitz, Maximilian F. Reiser, Christian Kupatt, Hans C. Becker

**Affiliations:** 1 Institute for Clinical Radiology, Hospital of the Ludwig Maximilian University of Munich, Munich, Germany; 2 Medical Clinic I – Cardiology, Hospital of the Ludwig Maximilian University of Munich, Munich, Germany; 3 HELIOS Klinikum Siegburg, HELIOS Kliniken GmbH, Siegburg, Germany; 4 Department of Cardiac Surgery, Hospital of the Ludwig Maximilian University of Munich, Munich, Germany; 5 DZHK (German Centre for Cardiovascular Research), Partner Site Munich Heart Alliance, Munich, Germany; S. G. Battista Hospital, Italy

## Abstract

**Background/Objectives:**

To evaluate the predictive value of CT-derived measurements of the aortic annulus for prosthesis sizing in transcatheter aortic valve implantation (TAVI) and to calculate optimal cutoff values for the selection of various prosthesis sizes.

**Methods:**

The local IRB waived approval for this single-center retrospective analysis. Of 441 consecutive TAVI-patients, 90 were excluded (death within 30 days: 13; more than mild aortic regurgitation: 10; other reasons: 67). In the remaining 351 patients, the CoreValve (Medtronic) and the Edwards Sapien XT valve (Edwards Lifesciences) were implanted in 235 and 116 patients. Optimal prosthesis size was determined during TAVI by inflation of a balloon catheter at the aortic annulus. All patients had undergone CT-angiography of the heart or body trunk prior to TAVI. Using these datasets, the diameter of the long and short axis as well as the circumference and the area of the aortic annulus were measured. Multi-Class Receiver-Operator-Curve analyses were used to determine the predictive value of all variables and to define optimal cutoff-values.

**Results:**

Differences between patients who underwent implantation of the small, medium or large prosthesis were significant for all except the large vs. medium CoreValve (all p’s<0.05). Furthermore, mean diameter, annulus area and circumference had equally high predictive value for prosthesis size for both manufacturers (multi-class AUC’s: 0.80, 0.88, 0.91, 0.88, 0.88, 0.89). Using the calculated optimal cutoff-values, prosthesis size is predicted correctly in 85% of cases.

**Conclusion:**

CT-based aortic root measurements permit excellent prediction of the prosthesis size considered optimal during TAVI.

## Introduction

Aortic valve stenosis is the most common acquired valve disorder and symptomatic forms have dismal outcomes when treated medically [1], [2]. For decades, surgical valve replacement has been the only curative treatment – however, due to comorbidities at the time of presentation up to one third of patients cannot undergo open heart surgery [3]–[5]. Transcatheter Aortic Valve Implantation (TAVI)/Transcatheter Aortic Valve Replacement (TAVR) is a novel, less invasive technique and is comparably safe even in patients with contraindications to surgery [6], [7]. Results of the randomized controlled PARTNER-B-cohort comparing TAVI to best medical therapy have shown substantial survival benefits after 12 and 24 months [8], [9]. In patients with a high surgical risk (PARTNER-A-cohort), TAVI was non-inferior to surgery after 12 months [10].

Unlike in surgical replacement, prosthesis sizing for TAVI significantly relies on imaging [11]. Imaging-derived measurements of the aortic root play the key role in patient and device selection. Transesophageal echocardiography and Multidetector CT-angiography (CTA) have been applied extensively in this regard [12]–[14]. Several studies have consistently demonstrated that the aortic annulus has an elliptic shape described by a long and short axis with a wide range of reported eccentricities [15], [16]. As a consequence, it is difficult to measure the true dimensions of the aortic annulus on the basis of a single plane obtained by 2D-echocardiography [17], [18].

There is initial evidence favoring CTA over echocardiography for prosthesis selection. Recently, Jilaihawi et al had demonstrated for the SAPIEN XT valve (Edwards Lifesciences) that annular-sizing on the basis of CT resulted in lower rates of paravalvular regurgitation than sizing on the basis of 2D TEE [17]. Similar results had previously been reported by Hayashida et al for patients having undergone implantation of the Corevalve (Medtronic) or Sapien XT valve (Edwards Lifesciences) [19].

Several questions remain as to how select the optimal prosthesis size on the basis of CT-derived annulus parameters and most authors use a fixed algorithm suggesting certain annulus diameter ranges for distinct prosthesis sizes. Recently, Binder et al reported that the application of a CT-based annulus area sizing algorithm prior to TAVI resulted in the reduction of paravalvular regurgitation compared with simply providing quantitative results for anatomical parameters [20].

In this study, we analyzed all patients (n = 351) who had undergone dedicated CT-angiography prior to TAVI at our institution. We report descriptive statistics for the key anatomic parameters of the aortic root, determine interobserver reproducibility for CT-derived measurements and analyze various anatomic variables for their predictive value for the selection of optimal device size. Suggestions are provided for optimal cutoff values for CT-based measurements.

## Methods

### 1. Patient Population

This analysis included patients with severe aortic valve stenosis who underwent a TAVI procedure at our institution between November 2007 and June 2012. Patients needed to have undergone CTA for the evaluation of aortic root anatomy within three months before TAVI. As all CT scans were performed as part of routine clinical workup and were analyzed anonymously, the institutional review board of the Faculty of Medicine of the Ludwig Maximilian University of Munich waived the necessity to obtain consent beyond routine clinical requirements. All patients gave written consent to an anonymous analysis of the acquired data.

**Figure 1:**
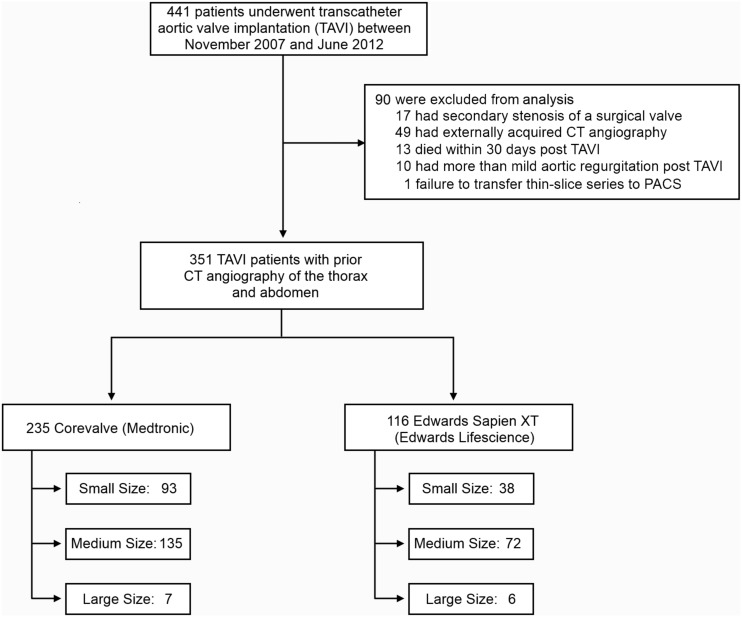
Inclusion chart for our analysis of 351 patients who underwent a successful TAVI procedure.

According to institutional policies patients with impaired renal function (glomerular filtration rate <30 ml/min), abnormal TSH-levels or a history of allergic reaction to iodine-containing contrast agents were excluded. After explicit education about the risks of iodinated contrast agents and exposure to x-rays, written informed consent was obtained (Figure 1).

### 2. CT Data Acquisition and Image Reconstruction

CT scans were performed either on a first-generation dual-source MDCT scanner (n = 49, Somatom Definition, Siemens Healthcare, Forchheim, Germany) or on a second-generation dual-source MDCT scanner (n = 302, Somatom Definition Flash, Siemens Healthcare) 3–90 days before TAVI. The ECG-signal was registered continuously throughout the scan. Images of the heart were acquired during diastole. Slice collimation was 2×64×0.6 mm (first generation) or 2×128×0.6 mm (second generation). Tube potential was 100 or 120 kV (depending on patient weight) and effective tube current-time product was 350–400 mAs/rotation. See Appendix S1 for detailed scan parameters.

In all patients, 90 ml of iomeprol 816.5 g/l (Imeron 400, Bracco Imaging, Milan, Italy) were administered via an ante-cubital vein at a flow-rate of 4 ml/s, followed by 100 ml of normal saline at the same flow rate. Contrast enhancement was controlled by bolus tracking within the ascending aorta.

Once intraluminal attenuation exceeded 150 HU, the table was repositioned for the desired scan range. Delay was 6 seconds (Somatom Definition) or 12 seconds (Somatom Definition Flash). Thereafter, the start of the scan was triggered by ECG automatically.

A medium-smooth convolution kernel was used to generate standard coronary CTA reconstructions (small field of view, slice thickness 0.75 mm, reconstruction increment 0.5 mm covering the entire heart). All series were pseudonymized and transferred to an external workstation.

### 3. CT Data Analysis

**Figure 2:**
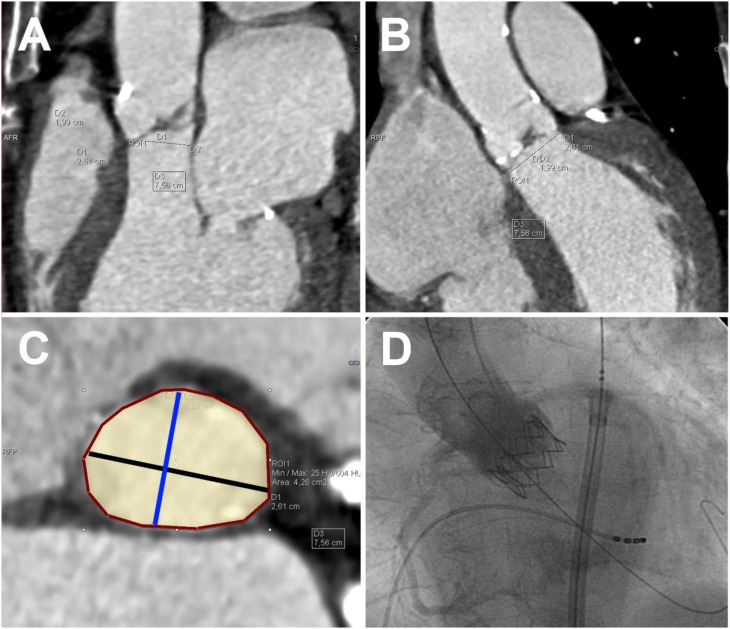
Examples of anatomic measurements at the aortic annulus performed for this study: isotropic small field-of-view CTA dataset of a 78 year old female patient with severe aortic stenosis, A) sagittal reformation and B) coronal reformation showing the orientation of the aortic annulus plane, C) double-oblique reformation of the aortic valve annulus, demonstrating the diameter of the long axis (black line), the diameter of the short axis (blue line), the annulus circumference (red polygonal), and annulus area (yellow shading). D) angiographic image after implantation of a 26 mm model of the Edward Sapien XT valve prosthesis demonstrates no paravalvular leakage.

Two experienced readers analyzed all series independently using commercially available software (Syngo Via VA20, Siemens Healthcare, Germany). Readers determined subjective image quality and contrast enhancement on 4-point Likert scales (4: best image quality/contrast enhancement, 1: poor image quality/contrast enhancement). Furthermore, both readers measured the size of the aortic annulus according to techniques suggested by various authors [12], [21]–[23]. Using multiplanar reformations, readers independently established the double-oblique plane defined by the three ‘hinge’ points (i.e. the most apical points of the valvular cusps, see Figure 2). On this plane, both readers manually determined the lengths of the long axis (LA), short axis (SA), circumference (C) and area (A) of the elliptical shape of the the aortic valve annulus (Figure 2). Three virtual diameters (*d_mean_, d_C_ and d_A_*) were derived from these parameters according to the following formulae:
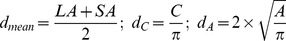



### 4. Clinical and procedural data

In all patients, TAVI was performed at the department of cardiology of this institution as part of routine clinical care. Patients were admitted at least 1 day prior to TAVI and transferred to a high-level intensive care unit for at least 24 hours afterwards. All patients underwent a routine transthoracic echocardiography exam within 7 days prior to TAVI.

Selection of valve type (CoreValve by Medtronic, Minneapolis, USA vs. Edward Sapien XT by Edwards Lifesciences, Irvine, CA, USA) and size was performed by the heart team members before and during TAVI. According to the standard operating procedures of this institution, an initial estimation of annulus size was performed based on all available imaging information. During the procedure, a balloon catheter with the estimated size was inflated at the annulus position and snug fit was evaluated by fluoroscopy and balloon pressures [24]–[26]. Depending on the appearance of the balloon and the recorded pressures, valve size was either confirmed or changed. In the latter case, a different balloon catheter was used and the changed size tested. Once optimal size had been established, the respective valve was implanted using the techniques recommended by manufacturers. Thereafter, conventional angiography was performed to test for aortic regurgitation, which was rated on a three point Likert scale (1: no, minimal or first degree regurgitation, 2: second degree regurgitation, 3: severe regurgitation).

### 5. Statistical Analysis

The D’Agostino-Pearson test was used to test for normal distribution of continuous variables. Continuous variables are reported as mean ± standard deviation when normally distributed, otherwise as median (interquartile range).

For categorical variables, Cohen’s kappa was used to address observer agreement and was reported with 95% confidence interval. For continuous variables, the intraclass correlation coefficient (ICC) was used [27].

Differences in proportions were assessed using the Chi-squared test. To test for differences in means of interval variables, the Student’s t-test (for independent or paired samples) was applied if variables followed normal distribution. For comparison of means of three or more variables, a one-way analysis of variance (ANOVA) was performed. If significant differences were found, variables were compared using the Tukey-HSD post-hoc test with application of Bonferroni corrections to avoid errors due to multiple testing.

To compare means in variables not following normal distribution, a t-test was used if normal distribution was approximated after logarithmic transformation. In all other cases, non-parametric tests were used: the Mann-Whitney-test for independent variables and the Wilcoxon signed-rank test for paired variables.

To identify cut-off points that allow optimal prediction of implanted valve size on the basis of the particular anatomic parameter, and to estimate the associated generalization performance, we applied a 10-fold nested cross-validation approach. Details regarding this approach are included in Appendix S1.

P-values of 0.05 or less were considered statistically significant unless otherwise stated. Data were analyzed using MedCalc (Version 9.3.0.0, MedCalc Software, Mariakerke, Belgium), SPSS (21.0, IBM, Armonk, USA) and R [28].

## Results

### 1. Patient Population

Between November 2007 and June 2012, 441 consecutive patients with high-grade aortic stenosis (mean AVA: 0.70 cm^2^, SD: 0.15 cm^2^) underwent a TAVI procedure at our institution. Of these, 17 patients were excluded as they presented with secondary stenosis of a surgical aortic valve prosthesis. Another 49 patients presented with externally acquired CT angiography datasets and were also excluded from this analysis. Of the remaining patients, 13 had died within 30 days after procedure and were not included into further analysis. 10 patients were excluded as they showed more than mild aortic regurgitation after valve implantation. Finally, one patient was excluded due to an error during archiving the small FOV (cardiac) series to PACS (Figure 1).

Of the remaining 351 patients, 235 had undergone implantation of a CoreValve prosthesis while in the remaining 116 patients the Edwards Sapien XT valve had been implanted. While differences in demographic parameters and preinterventional echocardiographic parameters between patients who underwent CoreValve vs. Edward Sapien XT valve implantation were not statistically significant, there was a difference in logarithmic Euro-Scores (19.6 [14.5; 26.7] vs. 16.5 [11.0; 23.4], p<0.01, see Table 1 for a summary of patient parameters) demonstrating a moderately less favorable risk profile in the CoreValve subgroup. In the entire cohort, no adverse events due to iv contrast administration during CT angiography were reported.

**Table 1:** Patient parameters, N(%), mean ± SD or median [interquartile range] and summary of implanted valve sizes.

	All Patients	CoreValveSubgroup	Edward SapienSubgroup	p
**n**	351 (100%)	235 (67%)	116 (33%)	
**Female Patients**	211 (58%)	132 (54%)	74 (62%)	0.18
**Age at scan (yrs)**	80.9±10.8	81.6±6.5	76.3±21.5	0.06
**Height (cm)**	165.7±8.9	165.5±13.9	163.2±18.2	0.125
**Weight (kg)**	72.9±14.7	72.6±15.0	72.1±17.0	0.83
**BMI (kg/m^2^)**	26.5±4.8	26.3±4.5	26.8±5.4	0.62
**Aortic Valve Area (cm^2^)**	0.70±0.15	0.69±0.16	0.72±0.13	0.21
**Pressure gradient (mmHg)**	66.9±22.4	67.3±23.8	66.0±19.6	0.65
**Ejection fraction (%)**	58.0±15.3	57.8±16.0	58.3±13.6	0.80
**Logistic EuroSCORE**	18.9 [13.4; 25.7]	19.6 [14.5; 26.7]	16.5 [11.0; 23.4]	<0.01
**Valve Sizes:**				
** Small Size**	131 (37%)	93 (40%)	38 (33%)	0.25
** Medium Size**	207 (59%)	135 (57%)	72 (62%)	0.44
** Large Size**	13 (4%)	7 (3%)	6 (5%)	0.52

### 2. Subjective image quality and contrast enhancement of CTA datasets

There was substantial interobserver agreement for both subjective overall image quality (kappa = 0.76 [0.69–0.82]) and contrast enhancement (kappa = 0.72 [0.67–0.77]). Therefore, values from reader one were used for further analysis. Mean subjective image quality was 3.66 (SD: 0.58) and mean contrast enhancement was 3.88 (SD: 0.43).

### 3. CT-based anatomical measures of the aortic root

Interobserver agreement was excellent for all parameters with ICC-values consistently above 0.80. Bland-Altman-Analysis revealed no relevant systematic differences (Table 2 and Appendix S2). Therefore, arithmetic means between both observers were calculated for all variables.

**Table 2:** Interobserver Agreement for anatomical measurements of the aortic root reported as Intraclass Correlation Coefficient (ICC) and parameters of Bland-Altman-Analysis.

	Diameter long axis	Diameter short axis	Diameter*_Mean_*	Annulus Circum-ference	Annulus Area
**ICC [95% CI]**	0.87 [0.84; 0.89]	0.86 [0.83; 0.88]	0.90 [0.88; 0.92]	0.92 [0.90; 0.93]	0.93 [0.92; 0.94]
**Mean difference**	0.00 cm	0.00 cm	0.00 cm	0.06 cm	0.03 cm^2^
**SD of differences**	0.14 cm	0.12 cm	0.20 cm	0.54 cm	0.6 cm^2^

Table 3 provides descriptive statistics on aortic valve measurements. Across all patients, length of the long axis and short axis were 2.70 [2.52; 2.90] and 2.05 [1.95; 2.25], respectively, and were slightly larger in the CoreValve subgroup than in the Edward Sapien subgroup (see Table 3). Median circumferences and median areas were 7.65 [7.28; 8.15] cm and 4.34 [3.90; 4.95] cm^2^. There was a small but significant difference in average annulus diameters derived from circumference (2.44 [2.32; 2.60] cm) vs. those derived from annulus area (2.35 [2.23; 2.51] cm, p<0.01).

**Table 3:** Primary anatomic parameters of the aortic annulus, N(%), mean ± SD or median [interquartile range] as well as “virtual diameters” derived from the mean of long axis and short axis, from annulus circumference and annulus area.

	All Patients	CoreValveSubgroup	Edward SapienSubgroup	p
**Diameter Long Axis [cm]**	2.70 [2.52; 2.90]	2.75 [2.54; 2.90]	2.65 [2.50; 2.85]	0.049
**Diameter Short Axis [cm]**	2.05 [1.95; 2.25]	2.10 [1.95; 2.28]	2.05 [1.90; 2.20]	<0.01
**Circumference [cm]**	7.65 [7.28; 8.15]	7.73 [7.30; 8.25]	7.55 [7.05; 8.00]	<0.01
**Area [cm^2^]**	4.34 [3.90; 4.95]	4.44 [3.98; 5.05]	4.19 [3.70; 4.75]	<0.01
**Diameter** ***_Mean_*** ** [cm]**	2.40 [2.25; 2.55]	2.42 [2.27; 2.58]	2.35 [2.22; 2.52]	<0.01
**Circumference- derived virtual** **diameter [cm]**	2.44 [2.32; 2.60]	2.46 [2.32; 2.63]	2.40 [2.24; 2.55]	<0.01
**Area-derived virtual** **diameter [cm]**	2.35 [2.23; 2.51]	2.38 [2.25; 2.54]	2.31 [2.17; 2.46]	<0.01

### 4. Annulus dimensions for different valve sizes

Of the 235 patients who underwent implantation of a CoreValve prosthesis, the 26 mm, 29 mm and 31 mm models were chosen in 93 (40%, 93/235), 135 (57%, 135/235) and 7 (3%, 7/235) cases. In 116 patients who received the Edward Sapien XT valve, the 23 mm, 26 mm and 29 mm were implanted in 38 (33%, 38/116), 72 (62%, 72/116) and 6 (5%, 6/118) cases.

**Figure 3:**
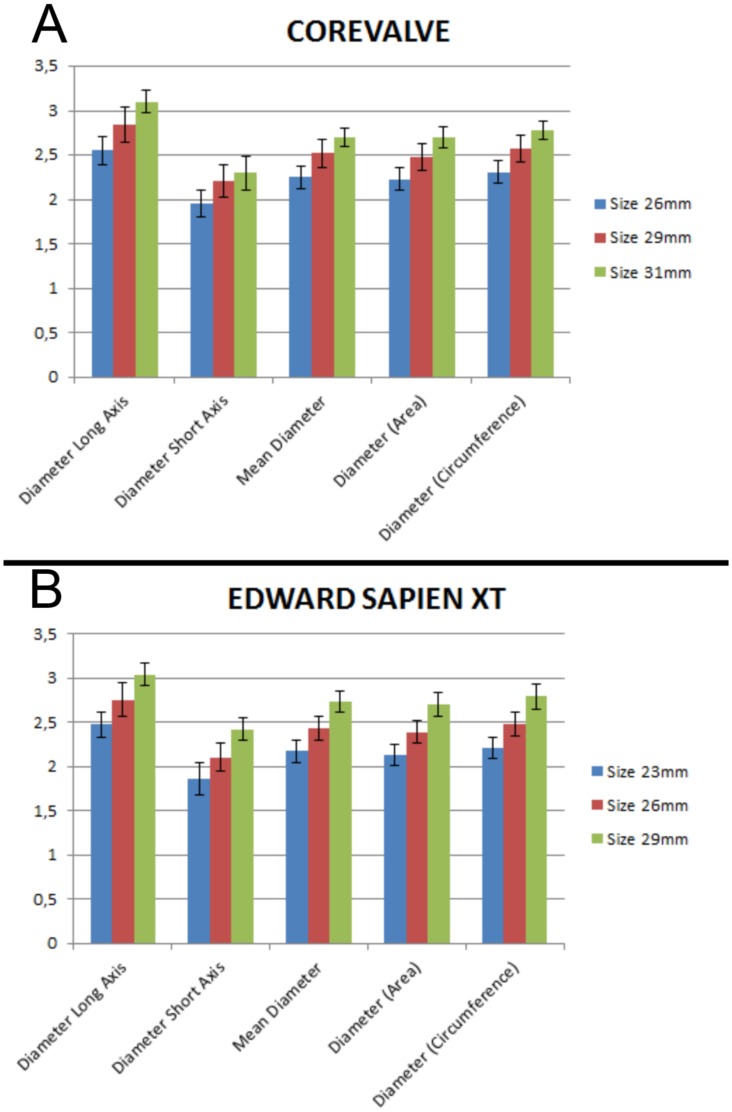
Summary chart displaying major anatomic parameters (length of long and short axis; circumference-derived and area-derived virtual diameter) in patients in whom the small, medium or large size model was considered optimal for A) the CoreValve valve and the B) Edward Sapien XT valve. Except for the large vs. medium CoreValve size, all differences were statistically significant (p<0.05).

Figure 3 provides details on long axis diameter, short axis diameter, circumference-derived and area-derived average annulus diameter measured for different sizes of CoreValve (Figure 3A) and Edward Sapien XT (Figure 3B). For all analyzed variables, one-way ANOVA showed significant differences between patients who underwent implantation of the small, middle or large valve (for the CoreValve: 26 mm, 29 mm, 31 mm; for the Edward Sapien XT: 23 mm, 26 mm, 29 mm, both p’s<0.01). In Tukey-HSD post-hoc tests, differences between all size subgroups were statistically significant except for patients who underwent implantation of the CoreValve 31 mm vs. 29 mm (p>0.05), which was most likely due to the relatively small number of patients in the 31 mm CoreValve subgroup (n = 7).

### 5. Predictive value of different variables for device size selection and optimal cutoff values

Using multi-class ROC-analysis, the predictive value of all anatomic parameters for the size of prosthesis considered optimal was evaluated. For both valve types, all five analyzed parameters showed high predictive value for optimal prosthesis size (AUC: 0.75–0.91, Table 4). In particular, for the Medtronic CoreValve the short axis had a somewhat lower predictive value (multi-class AUC: 0.75) while there were no differences in predictive value between diameters of long axis, mean diameter, annulus area or annulus circumference (multi-class AUC’s: 0.81, 0.80, 0.88, 0.9, p>0.05, Appendix S3). For the Edwards Sapien XT valve, differences in predictive value between the different parameters also did not reach statistical significance (multi-class AUC’s: 0.83, 0.80, 0.88, 0.88, 0.89, p>0.05, Appendix S4). Application of the proposed cutoff-values in our sample of 351 patients results in the correct classification of 81.5% (286/351), 85.5% (300/351) and 82.9% (291/351) of cases if annulus circumference, annulus area or mean diameter are used.

**Table 4:** Multi-Class AUC’s as measures for the predictive value of the respective anatomic parameters for the valve size considered optimal by the implantation team.

	PARAMETER	OPTIMAL CUTOFFSMALL vs. MEDIUM	OPTIMAL CUTOFFMEDIUM vs. LARGE	MULTI-CLASS AUC
**MEDTRONIC COREVALVE**	Diameter Long Axis	2.70 cm	3.04 cm	0.8133
	Diameter Short Axis	2.05 cm	2.29 cm	0.7478
	Diameter*_Mean_*	2.35 cm	2.70 cm	0.7974
	Annulus Area	4.30 cm^2^	5.72 cm^2^	0.8812
	Annulus Circumference	7.65 cm	8.60 cm	0.9146
**EDWARDS SAPIEN** **XT**	Diameter Long Axis	2.55 cm	3.05 cm	0.8347
	Diameter Short axis	1.95 cm	2.30 cm	0.7995
	Diameter*_Mean_*	2.25 cm	2.55 cm	0.8793
	Annulus Area	3.85 cm^2^	5.35 cm^2^	0.8823
	Annulus Circumference	7.15 cm	8.20 cm	0.8884

Optimal cutoff values for the selection of the small, medium or large prosthesis size, defined as cutoff values that result in highest predictive accuracy.

## Discussion

Transcatheter aortic valve implantation (TAVI) has been developed as a treatment strategy for patients with severe aortic stenosis who are at high risk or not eligible for heart surgery [29]. Several studies have demonstrated that MDCT is an ideal imaging modality prior to TAVI, providing isotropic datasets of the aortic root that can be reformatted in any spatial orientation [30]–[32]. There is some evidence favoring CT-derived annulus sizing over transthoracic echocardiography, particularly in regard to the incidence of postinterventional aortic regurgitation [17], [19].

Several methods have been proposed to reduce the shape of the aortic annulus to a single measure for prosthesis sizing: calculation of the arithmetic mean between long and short axis [21], measurement of the length of the annulus circumference [12] or quantification of the annulus area [22], [23]. To date, no consensus has been established on what the best technique is.

Binder et al have recently demonstrated for the Sapien XT valve that the recommendation of a particular prosthesis size on the basis of CT data results in more favorable outcomes than simply providing the annulus parameters [20]. In this regard however, uncertainty remains as to what the optimal size of prosthesis is for a given set of aortic annulus parameters.

In this analysis of over 350 patients who had undergone CT angiography prior to TAVI, we performed extensive anatomic measurements of the aortic root. Our results confirm previous analyses of Halpern et al [14], Gurvitch et al [12] and Delgado et al [33], since all relevant anatomic parameters can be measured in virtually all patients with excellent agreement between two experienced observers. Similar to the study by Gurvitch, observer agreement was highest for aortic annulus circumference and area (ICC’s = 0.92, 0.93), even though in our study differences to observer agreement for long and short axis measurements were not significant.

In all patients included in this analysis, optimal prosthesis size was determined with the help of a sizing balloon catheter inflated during the implantation procedure [24]–[26]. Furthermore, all patients in whom this method might not have resulted in the selection of the correct prosthesis size – i.e. patients with more than mild regurgitation after TAVI (n = 10) and patients who died within 30 days after implantation (n = 13) – were deliberately excluded. No patient had to be excluded due to open heart surgery within 30 days. According to best professional judgment, prosthesis sizing was adequate in the remaining 351 patients. Thus, our data permit the unique opportunity to analyze the relation between different anatomic variables of the aortic root and the device size ultimately considered optimal. Even more importantly, the discriminatory power of different anatomic variables to predict prosthesis size as well as optimal cutoff values can be calculated.

Our results demonstrate that for all analyzed anatomic variables there are differences between patients who underwent implantation of the small, medium or large valve. Our results are both in line with but also extend those of Jilaihawi et al [17] and Delgado et al [34], demonstrating that on the basis of high-resolution MDCT datasets of the aortic root, the optimal valve size can be predicted. While in principle all evaluated anatomic parameters can be used for prosthesis sizing, the most favorable combination of high observer agreement and predictive value are observed for mean diameter, annulus area and annulus circumference (with multi-class AUC’s of 0.88 and 0.91). Optimal thresholds for the selection of the small, medium or large size are given in Table 4. The use of these cutoff values in our cohort results in the correction prediction of valve size in 85% (300/351) of cases. In the remaining 15% (51/351), the application of a sizing balloon would result in a change of device size. Importantly, our 2-threshold-model does not take into account that a considerable number of patients will be suitable candidates for two valve sizes. Inclusion of the extent of aortic valve calcifications might further increase the predictive accuracy of our model.

Our single-center retrospective study has several limitations: first, the heart team members involved in valve implantation were not blinded towards the routine radiological reports derived from the CT dataset. While this would have been favorable from a scientific perspective, we feel that the information obtained from high-resolution CT angiography datasets prior to TAVI is of so essential a nature that withholding it would be clearly unethical. Furthermore, cardiologists integrated all available information regarding valve size, including transthoracic and transesophageal echocardiography and aortic angiography usually conducted before the TAVI procedure. In every single case, the presumed optimal valve size was simulated with a balloon catheter and changed if the size of the inflated balloon and the aortic annulus did not display the estimated ratio during aortography.

Second, we did not compare our measurements with a reference imaging modality such as 3D-echocardiography or MR angiography. However, our reference standard is the size of the implanted valve in those patients in whom – according to best professional judgment – prosthesis size turned out to be reasonably selected. For the purpose of this analysis we consider this outcome-oriented reference standard superior.

Third, we used only diastolic CT acquisitions to measure aortic annulus dimensions. However, it still is a matter of debate if systolic reconstructions are necessary for a reliable sizing of the aortic valve annulus: while some authors propose systolic reconstructions to avoid undersizing [35], [36], others report no difference [37] or no substantial difference [38] between systolic and diastolic diameters. However, since we correlate our diastolic measures of annulus parameters with the prosthesis size considered optimal during implantation, a potential difference between systolic and diastolic dimensions is not a major concern; our results should rather be construed as a contribution to a prosthesis sizing algorithm from diastolic annulus parameters.

Fourth, we do not perform an analysis of the patients in whom valve implantation was not successful. However, the main intention of our manuscript is to perform a rigorous analysis of the dependencies between anatomical parameters of the aortic annulus and the implanted valve size for patients in whom the procedure was performed successfully. In that sense, we do not attempt to prove that an inadequate selection of prosthesis size increases the risk of a negative procedural outcome.

Finally, while our analysis suggests an ideal strategy to predict the appropriate valve size, we cannot answer the question how this strategy would perform in a prospective study context. However, the rate of excluded patients is comparatively small: only 23 patients had to be excluded due to either death within 30 days after the TAVI procedure (n = 13) or due to the development of more than mild aortic regurgitation (n = 10). Therefore, we are confident that our approach would permit a correct estimation of correct valve size in the vast majority of cases.

In conclusion, our analysis of 351 patients who successfully underwent TAVI shows that the valve size ultimately considered optimal can be predicted on the basis of CT-derived anatomic parameters of the aortic root. Mean diameter, annulus circumference and annulus area appear to have equally high reproducibility and predictive accuracy for both the Medtronic CoreValve and the Edwards Sapien XT prostheses. It remains to be shown that this approach holds up its predictive potential in a prospective study context.

## Supporting Information

Appendix S1 **Supplemental methods section including details on CT data acquisition, image reconstruction and statistical methods.**
(DOCX)

Appendix S2 **Supplemental scatter plots and linear regression lines for the analysis of interobserver agreement.**
(DOCX)

Appendix S3 **Extended results regarding the ANOVA analysis and post-hoc tests of differences in anatomic parameters of patients who underwent implantation of the large, middle or small version of either valve.**
(DOCX)

Appendix S4 **Extended results regarding the analysis of differences in predictive values of the various anatomic parameters for the valve size considered optimal by the TAVI team.**
(DOCX)
