# Supplementary material for: CT-Angiography–Based Evaluation of the Aortic Annulus for Prosthesis Sizing in Transcatheter Aortic Valve Implantation (TAVI)–Predictive Value and Optimal Thresholds for Major Anatomic Parameters
Source: PLoS One. 2014 Aug 1;9(8):e103481. doi: 10.1371/journal.pone.0103481 (PMC4118882; doi:10.1371/journal.pone.0103481)
Supplement: Appendix S1 — Supplemental methods section including details on CT data acquisition, image reconstruction and statistical methods. (DOCX) [file pone.0103481.s001.docx]

**Appendix S1:**

**Details regarding the materials and methods section:**

Details on CT Data Acquisition and Image Reconstruction: (Materials and Methods, Section 2)

In patients who were scanned on the first-generation dual-source scanner a prospectively ECG-triggered acquisition protocol with data acquisition during diastole (65% of the RR interval) was chosen with the scanrange confined to the heart. Collimation was 2 x 32 x 0.6 mm with a z-flying focal spot, resulting in a slice collimation of 2 x 64 x 0.6 mm. Gantry rotation time was 330 ms. Tube potential was selected according to patient weight (100 kV if patient weight ≤ 85 kg, otherwise 120 kV). Effective tube current-time product was 400 mAs.

On the second generation dual-source MDCT a high-pitch protocol was applied (pitch: 3.2). Tube potential was adjusted to patient weight, either manually (n = 175, 100 kV if weight ≤ 85 kg, otherwise 120 kV) or automatically on the basis of the topogram (n = 127, Care-kV([18](#_ENREF_18)), Siemens Healthcare). The remaining scan parameters were as follows: collimation: 2 x 64 x 0.6 mm with z-flying focal spot, resulting in a slice collimation of 2 x 128 x 0.6 mm; gantry rotation time: 280 ms; pitch: 3.2; an online tube current modulation program was used for all scans (Care DOSE 4D, Siemens Healthcare) with a reference tube current-time product set to 350 mAs per rotation. A craniocaudal scan direction was chosen, with image acquisition of the heart beginning at 60% of the RR interval. Scan range was set to include the entire body trunk to the level of the proximal femoral arteries.

Details on the statistical methods: (Materials and Methods, Section 5)

Cut-off points were optimized as part the inner CV-loop over the range of measured values for the respective parameter using steps of 0.05 (i.e. 111 values in the case of “long axis diameter”). Cut-off points were chosen to minimize the absolute difference between observed and predicted implanted size. This difference was inversely weighted by the square root of group sizes, in order to account for their unequal distribution across patients. The cut-off points most frequently selected during the inner CV-loop were then used to predict generalization performance in the outer CV-loop. This procedure was repeated for all CV folds and final cut-off points determined as those most frequently selected. Performance was estimated as the mean multi-class ROC across CV-folds according to Hand et al[1].

We have performed all pairwise comparisons between parameters and their optimal cut-off points to evaluate differences in predictive value. For this purpose, linear mixed models were applied using the difference between predicted and real valve size as outcome variable, parameter type as fixed and subject indicators as random factors (using R's nlme package). Subsequently, we evaluated all pairwise comparisons and adjusted for multiple hypotheses testing using the method of Tukey (multcomp package).

1. Hand DJ, Till RJ (2001) A simple generalisation of the area under the ROC curve for multiple class classification problems. Mach Learn 45: 171–186.
