# Supplementary material for: CT-Angiography–Based Evaluation of the Aortic Annulus for Prosthesis Sizing in Transcatheter Aortic Valve Implantation (TAVI)–Predictive Value and Optimal Thresholds for Major Anatomic Parameters
Source: PLoS One. 2014 Aug 1;9(8):e103481. doi: 10.1371/journal.pone.0103481 (PMC4118882; doi:10.1371/journal.pone.0103481)
Supplement: Appendix S2 — Supplemental scatter plots and linear regression lines for the analysis of interobserver agreement. (DOCX) [file pone.0103481.s002.docx]

**Appendix S2:**

Analysis of interobserver agreement: Scatter plots and regression lines of measurements of various aortic annulus parameters by both observers.

OBS1: observer 1, OBS2: observer 2.

1. **Diameter Long Axis:**

1. **Diameter Short Axis:**

1. **Annulus Area:**

1. **Annulus Circumference:**

1. **Mean Diameter:**
