# Supplementary material for: CT-Angiography–Based Evaluation of the Aortic Annulus for Prosthesis Sizing in Transcatheter Aortic Valve Implantation (TAVI)–Predictive Value and Optimal Thresholds for Major Anatomic Parameters
Source: PLoS One. 2014 Aug 1;9(8):e103481. doi: 10.1371/journal.pone.0103481 (PMC4118882; doi:10.1371/journal.pone.0103481)

**Appendix S3:**

**Comparison of Annulus parameters for patients who underwent implantation of the large, middle or small valve model:**

1. **Medtronic Corevalve:**

One-way ANOVA:

**
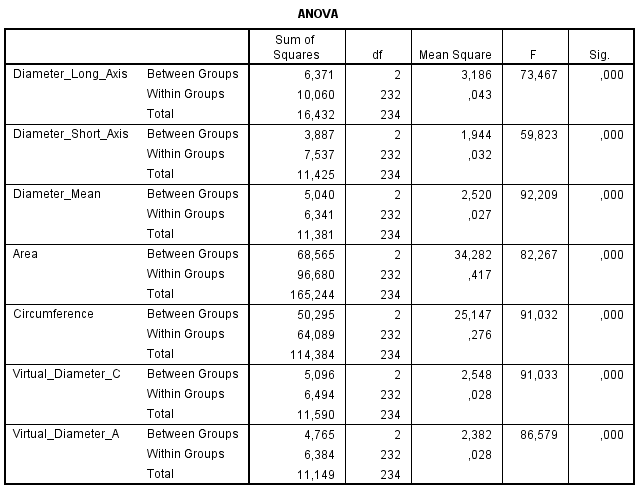
**

Tukey HSD Tests:

**
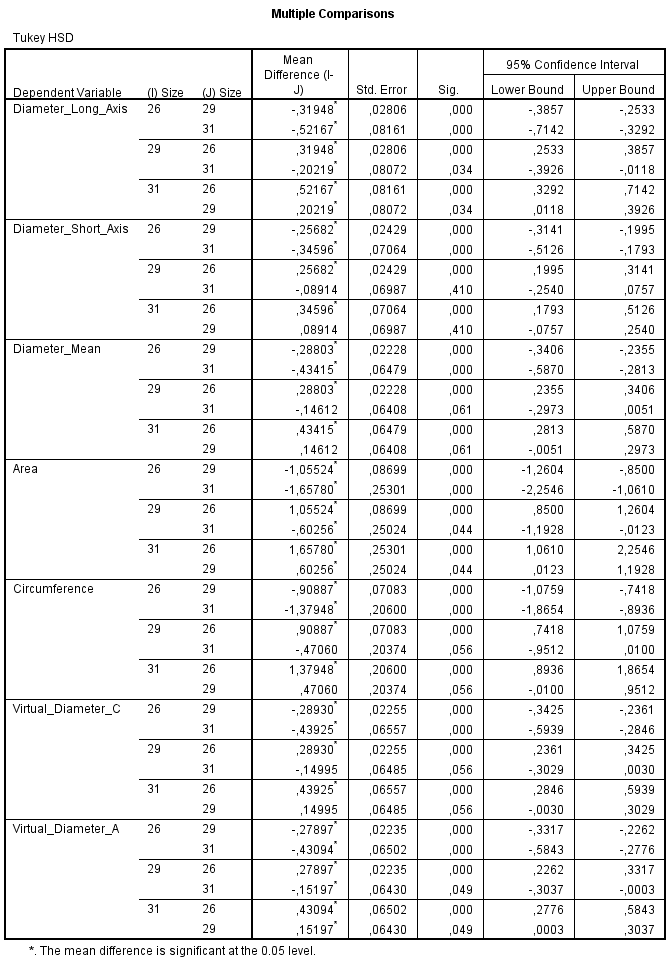
**

1. **Edward Sapien XT:**

One-way ANOVA:


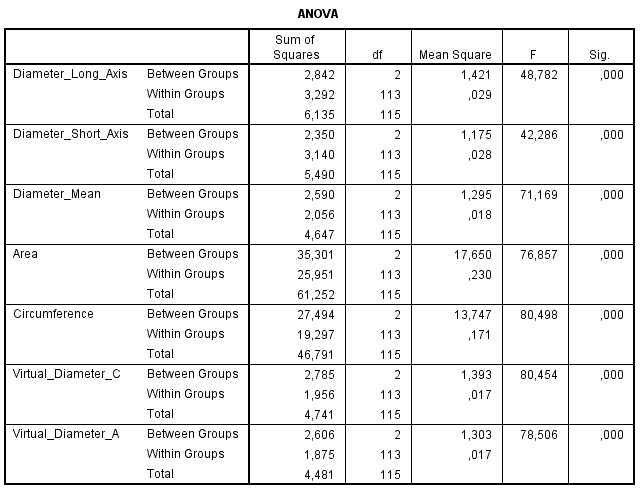


Tukey HSD Tests:


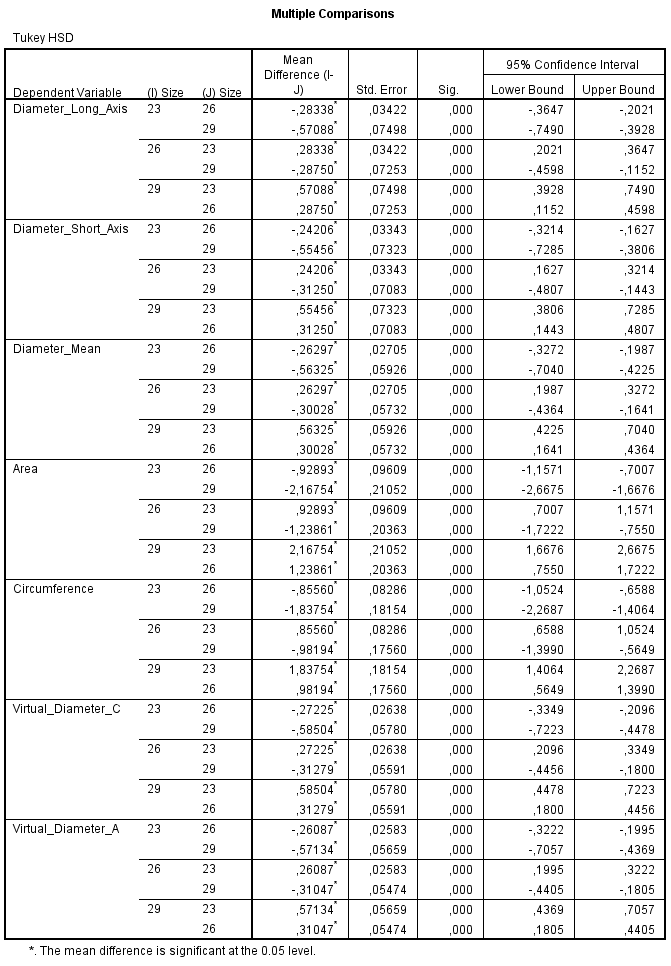

Supplement: Appendix S3 — Extended results regarding the ANOVA analysis and post-hoc tests of differences in anatomic parameters of patients who underwent implantation of the large, middle or small version of either valve. (DOCX) [file pone.0103481.s003.docx]
