# Supplementary figures and images for: CT-Angiography–Based Evaluation of the Aortic Annulus for Prosthesis Sizing in Transcatheter Aortic Valve Implantation (TAVI)–Predictive Value and Optimal Thresholds for Major Anatomic Parameters
Source: PLoS One. 2014 Aug 1;9(8):e103481. doi: 10.1371/journal.pone.0103481 (PMC4118882; doi:10.1371/journal.pone.0103481)

**Appendix S4:**

**Test for differences in predictive value of analyzed anatomical parameters**


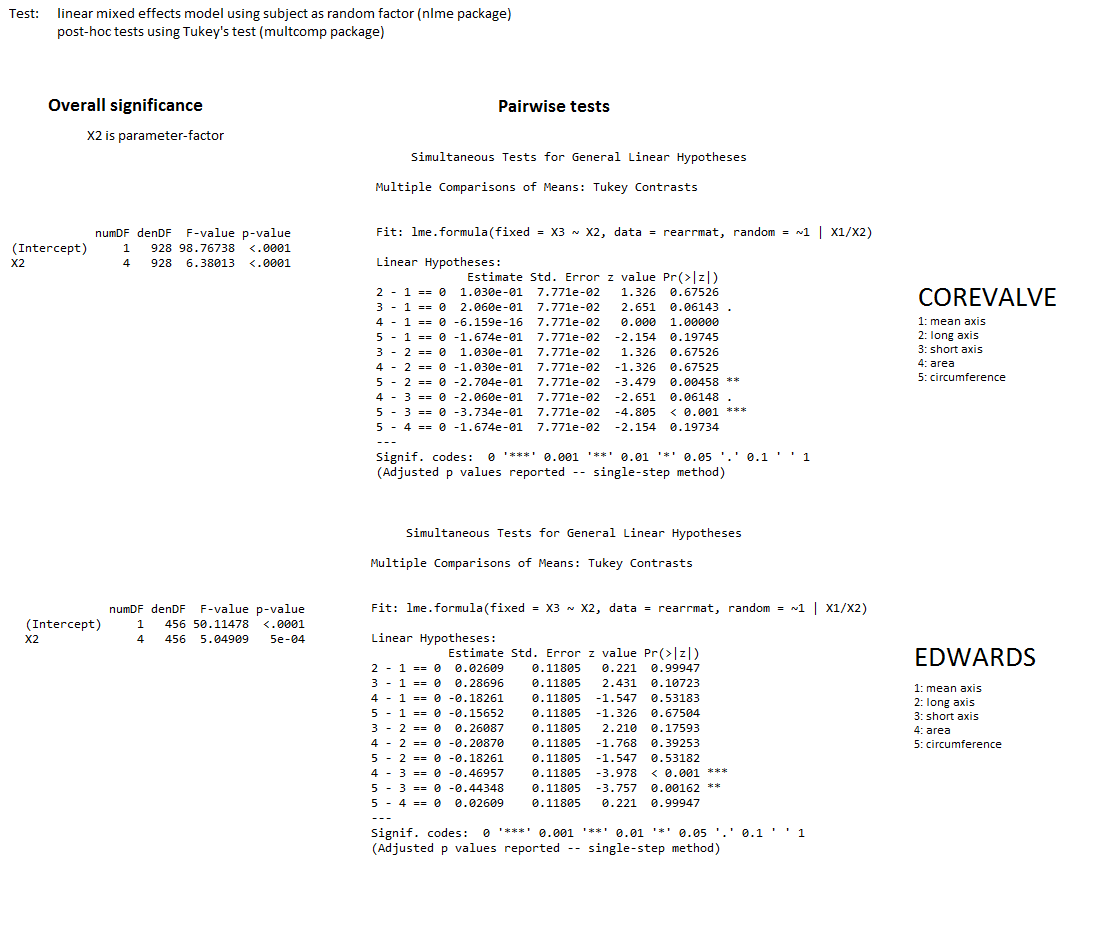

Supplement: Appendix S4 — Extended results regarding the analysis of differences in predictive values of the various anatomic parameters for the valve size considered optimal by the TAVI team. (DOCX) [file pone.0103481.s004.docx]
